# Diversity of *Anaplasma* and novel *Bartonella* species in *Lipoptena fortisetosa* collected from captive Eld’s deer in Thailand

**DOI:** 10.3389/fvets.2023.1247552

**Published:** 2023-09-14

**Authors:** Wittawat Wechtaisong, Chalida Sri-in, Kritsada Thongmeesee, Nichapat Yurayart, Chatlada Akarapas, Ganyawee Rittisornthanoo, Natcha Bunphungbaramee, Natthanicha Sipraya, Lyric C. Bartholomay, Umaporn Maikaew, Piyaporn Kongmakee, Arpussara Saedan, Sonthaya Tiawsirisup

**Affiliations:** ^1^Animal Vector-Borne Disease Research Unit, Veterinary Parasitology Unit, Department of Veterinary Pathology, Faculty of Veterinary Science, Chulalongkorn University, Bangkok, Thailand; ^2^Veterinary Pathobiology Graduate Program, Faculty of Veterinary Science, Chulalongkorn University, Bangkok, Thailand; ^3^Faculty of Veterinary Science, Chulalongkorn University, Bangkok, Thailand; ^4^Department of Pathobiological Sciences, School of Veterinary Medicine, University of Wisconsin-Madison, Madison, WI, United States; ^5^Khao Kheow Open Zoo, Zoological Park Organization of Thailand, Chon Buri, Thailand; ^6^Animal Conservation and Research Institute, Zoological Park Organization of Thailand, Bangkok, Thailand

**Keywords:** prevalence, *Anaplasma*, *Bartonella*, *Lipoptena fortisetosa*, Eld’s deer, Thailand

## Abstract

*Lipoptena* insects are important ectoparasites of cervids and may affect humans that are incidentally bitten. The presence of zoonotic pathogen DNA, such as *Anaplasma*, and *Bartonella*, raises the importance of *Lipoptena* insects in veterinary and human medicine. Eld’s deer (*Rucervus eldii thamin*), an endangered wild ruminant in Thailand, are bred and raised in the open zoo. The semi-wild zoo environment suggests ectoparasite infestation and potential risk for mechanical transmission of pathogens to visitors, zoo workers, or other animals. However, epidemiology knowledge of pathogens related to endangered wild ruminants in Thailand is limited. This study aims to determine the prevalence and diversity of *Anaplasma* and *Bartonella* in the *L. fortisetosa* collected from captive Eld’s deer in Chon Buri, Thailand. Of the 91 *Lipoptena* DNA samples obtained, 42 (46.15%) and 25 (27.47%) were positive for *Anaplasma* and *Bartonella* by molecular detection, respectively. Further, 42 sequences of *Anaplasma* (4 nucleotide sequence types) showed 100% identity to those detected in other ruminants and blood-sucking ectoparasites. Twenty-five sequences of *Bartonella* (8 nucleotide sequence types) showed 97.35–99.11% identity to the novel *Bartonella* species from sika deer and keds in Japan. Phylogenetic trees revealed *Anaplasma* sequences were grouped with the clusters of *A. bovis* and other ruminant-related *Anaplasma*, while *Bartonella* sequences were clustered with the novel *Bartonella* species lineages C, D, and E, which originated from Japan. Interestingly, a new independent lineage of novel *Bartonella* species was found in obtained specimens. We report the first molecular detection of *Anaplasma* and *Bartonella* on *L. fortisetosa*, which could represent infectious status of captive Eld’s deer in the zoo. Wild animals act as reservoirs for many pathogens, thus preventive measures in surrounding areas should be considered to prevent pathogen infection among animals or potential zoonotic infection among humans.

## 1. Introduction

Deer keds of the genus *Lipoptena* spp. (Diptera: Hippoboscidae) are hematophagous insects that infest mammals (1). The insects become wingless after finding a suitable host and attach to a single host throughout their life span (2–4). Of over 30 species of *Lipoptena* insects worldwide, *L. fortisetosa* along with *L. cervi*, *L. depressa*, and *L. mazamae* are the most prevalent and threaten to wildlife, livestock, and pets (5–8). *Lipoptena fortisetosa* are found on sika deer (*Cervus nippon*), roe deer (*Capreolus capreolus*), and red deer (*Cervus elaphus*) in many countries and incidentally found on dogs (2, 9–12). In addition, humans can be bitten by *Lipoptena* insects (13). Several molecular epidemiological studies show *L. fortisetosa* harbors DNA of various pathogens, including *Anaplasma phagocytophilum*, *Babesia* spp., *Bartonella* spp., *Borrelia* spp., *Coxiella*-like endosymbionts, *Francisella tularensis*, *Mycoplasma* spp., *Rickettsia* spp., and *Theileria* spp. (14–16).

The genus *Anaplasma* includes intracellular gram-negative bacteria transmitted by ixodid ticks (17). Several *Anaplasma* spp., such as *A. marginale*, *A. centrale*, *A. ovis*, and *A. bovis*, are obligate bacteria parasitizing blood cells of many ruminants, while *A. platys* is mainly a pathogen of dogs (17). *Anaplasma phagocytophilum* is a pathogenic bacterium of a wide range of hosts, including humans and domestic and wild animals (18). In addition, *A. phagocytophilum* has been detected worldwide in wild ruminants and their ectoparasites (19–23). Although the role of wildlife in circulation of *Anaplasma* spp. is yet to be clearly defined, several species of wild ruminants are considered important reservoirs (24). In Thailand, studies found evidence of *A. platys* and *A. bovis* detection in *Dermacentor auratus* ticks collected from sambar deer (*Cervus unicolor*) (25). Because *D. auratus* ticks are found on humans in Thailand (26, 27), humans infected with *Anaplasma* bacteria via infected tick bites in addition to other deer ectoparasites is also possible.

*Bartonella* spp. are intra-erythrocytic gram-negative bacteria mainly transmitted among hosts by arthropod vectors, such as cat fleas (*Ctenocephalides felis*), body lice (*Pediculus humanus*), and sand flies (*Lutzomyia verrucarum*) (28–30). There are 45 *Bartonella* spp./subspp. Detected or isolated from various animals (31). *Bartonella schoenbuchensis*, *B. capreoli*, and *B. bovis* are detected in wild ruminants in several countries, which are strongly suspected to be transmitted by *Lipoptena* spp. (32–39). In Thailand, the novel *Bartonella* spp. was detected and isolated from captive Rusa deer (*Rusa timorensis*) blood samples (40). Since no ectoparasites are found on these deer, further studies are needed to determine whether ectoparasites transmit *Bartonella* among deer throughout Thailand. For zoonotic issues, human cases of bartonellosis caused by ruminant-related species, *B. schoenbuchensis* and *B. melophagi*, have been previously reported (41, 42). These findings highlight that although *Bartonella* bacterial infection in animals does not result in serious diseases, this wide range of infected animals could be a reservoir for potential zoonotic infection.

Khao Kheow Open Zoo is located within a wildlife sanctuary in Chon Buri province, eastern Thailand. Several endangered wildlife, as well as Eld’s deer (*Rucervus eldii thamin*), are bred and raised in the open zoo to increase population numbers. Here, wild animals can freely roam the open zoo and sanctuary areas, increasing the possibility of pathogen transmission among wild animals. Recent evidence shows ruminant-related blood pathogens, including *Anaplasma*, *Babesia*, *Ehrlichia*, and *Theileria*, in various species of ticks in this surrounding environment (43). Moreover, Tiawsirisup et al. (44) reported the presence of *L. fortisetosa* on Eld’s deer with *Theileria capreoli* and *T. cervi* in these insects in Thailand. Although DNA presence does not guarantee pathogen transmission, it may highlight the potential risk for mechanical transmission of pathogens to humans and healthy animals via bites of infected ectoparasites. Currently, knowledge surrounding epidemiology of pathogens related to endangered wild ruminants in Thailand is limited. This study aims to determine the prevalence and diversity of *Anaplasma* and *Bartonella* in the ectoparasite collected from captive Eld’s deer. Our findings may be used to understand the current status of pathogens among Eld’s deer and their ectoparasite, formulate animal welfare policies, and provide valuable information to prevent and control pathogens related to endangered wildlife species in the country.

## 2. Materials and methods

### 2.1. Background of *Lipoptena fortisetosa* specimens

From May to November 2021, 91 blood-sucking insects were collected from 12 Eld’s deer at the wildlife animal hospital, Khao Kheow Open Zoo. The Eld’s deer were admitted to the hospital for various reasons, such as disease diagnosis or regular health examination. Insect sample collection was done by veterinarians and zoo staff during an anesthetized stage of animals. Each specimen was kept in a microcentrifuge tube with RNA stabilization solution and transported to the Parasitology Unit, Faculty of Veterinary Science, Chulalongkorn University, for morphological identification by using a taxonomic key (45). All specimens were identified as *L. fortisetosa* (44). In addition, 38 males and 53 females were also defined during morphological identification.

DNA was extracted from each *Lipoptena* specimen using the IndiSpin Pathogen Kit (Indical Bioscience, Germany), according to the manufacturer’s instructions. For molecular identification of *Lipoptena* specimens, we examined DNA samples using PCR assay with primers LCO1490 and HCO2198 (46). PCR mixture and condition were described by Tiawsirisup et al. (44) and the product size was 658 bp, which was confirmed using DNA sequencing. Using the nucleotide BLAST tool, all representative and validated sequences showed the closest similarity (94.28–94.45%) to *L. fortisetosa* (OL850869) from China. Moreover, *L. fortisetosa* can be classified into two clades: “clade I” based on sequences already deposited in the GenBank database and “clade II” based on sequences of *Lipoptena* specimens collected in Thailand (44).

### 2.2. *Anaplasma* and *Bartonella* detection

All DNA samples were used for *Anaplasma* and *Bartonella* detection using the PCR assay. Primers EHR16SD and EHR16SR were used to amplify a 345 bp segment of the 16S rRNA gene of Anaplasmataceae members (47). Primers BhCS781p and BhCS1137n were used to amplify a 380 bp segment of the citrate synthase gene (*gltA*) of *Bartonella* spp. (48). The PCR mixture was performed in a 25 μL reaction volume containing a DNA template, 10x PCR buffer (KOD One, TOYOBO Co., Ltd., Japan), 10 μM of forward and reverse primers, and sterile distilled water. PCR conditions were adapted by following the manufacturer’s instructions for PCR buffer and annealing temperatures were followed according to relevant studies (47, 48). DNA from *A. marginale* and *B. henselae* isolates (positive control) and distilled water (negative control) were used as controls for the PCR assay. The *Anaplasma* and *Bartonella* PCR-positive products from *Lipoptena* specimens were purified using a GenepHlow Gel/PCR cleanup kit (Geneaid Biotech Ltd., Taiwan) and sent for nucleotide sequencing (U2Bio Co., Ltd., South Korea).

### 2.3. Nucleotide sequence and statistical analyses

Forty-two sequences from *Anaplasma* PCR-positive and 25 sequences from *Bartonella* PCR-positive samples were analyzed for the closest similarity with reference nucleotide sequences in the GenBank database using the NCBI nucleotide BLAST tool. All sequences were validated, aligned, and compared for genetic similarity using MegAlign (DNASTAR, Inc., United States). The number of nucleotide sequence types (ntSTs) of *Anaplasma* and *Bartonella* sequences were analyzed using DnaSP version 6.12.03 (49).

We analyzed the best-fit models for constructing phylogenetic trees using the Find Best DNA/Protein Model in MEGA X. Phylogenetic trees were generated using MEGA X with the maximum likelihood (ML) algorithm on the Kimura 2-parameter model plus gamma distribution (K2 + G) for *Anaplasma* sequences and Tamura-Nei parameter model plus gamma distribution (TN93 + G) for *Bartonella* sequences applied bootstrap method with 1,000 replications. ntST networks were constructed using the Median-joining (MJ) network in PopART version 1.7 (50, 51).

Pathogen infection rates in different genders of *Lipoptena* specimens were calculated and compared using Fisher’s exact test and *p* < 0.05 was considered statistically significant (GraphPad Prism 8.4.2 software, CA).

## 3. Results

### 3.1. *Anaplasma* and *Bartonella* detected in *Lipoptena* specimens

The PCR results showed that 46.15% (42/91) and 27.47% (25/91) of *L. fortisetosa* harbored *Anaplasma* and *Bartonella* DNA, respectively (Table 1). Based on the collecting date, *Lipoptena* specimens collected in June 2021 showed the highest prevalence of *Anaplasma* infection (66.67%; 24/36), while specimens collected in May 2021 showed the highest prevalence of *Bartonella* infection (39.28%; 11/28) and co-infection (28.57%; 8/28) (Table 1). However, no *Anaplasma* and *Bartonella* DNAs were detected from *Lipoptena* specimens collected in November 2021 (Table 1). *Anaplasma* infection rate in female specimens (47.16%; 25/53) was higher than in males (44.73%, 17/38; *p* = 0.8346). In addition, we also found a higher *Bartonella* infection rate in female specimens (33.96%, 18/53) than in males (18.42%, 7/38; *p* = 0.1525). Of the 91 specimens, 11 (12.08%) were co-infected with *Anaplasma* and *Bartonella* spp. (Table 1).

**Table 1:** Prevalence of *Anaplasma* and *Bartonella* spp. infection in *Lipoptena fortisetosa* detected by PCR.

Variables	Number of samples (*N* = 91)	Prevalence of infections (infected/tested samples)		
		*Anaplasma* spp.	*Bartonella* spp.	Co-infection (*Anaplasma* + *Bartonella*)
**Collecting date**				
May 2021	28	57.14% (16/28)	39.28% (11/28)	28.57% (8/28)
June 2021	36	66.67% (24/36)	27.78% (10/36)	8.33% (3/36)
August 2021	16	6.25% (1/16)	25.00% (4/16)	0% (0/0)
September 2021	3	33.33% (1/3)	0% (0/0)	0% (0/0)
November 2021	8	0% (0/0)	0% (0/0)	0% (0/0)
Total	91	46.15% (42/91)	27.47% (25/91)	12.08% (11/91)
**Gender**				
Male specimens	38	44.73% (17/38)	18.42% (7/38)	7.89% (3/38)
Female specimens	53	47.16% (25/53)	33.96% (18/53)	15.09% (8/53)

### 3.2. Genetic and BLAST analyses of *Anaplasma* and *Bartonella* detected in *Lipoptena* specimens

Among 67 validated sequences in this study, 42 sequences (primer cut; 305 bp) were from *Anaplasma* PCR-positive samples, while the other 25 were from *Bartonella* PCR-positive (primer cut; 337 bp) samples (Table 2). We aligned and compared the validated sequences of each pathogen, then grouped these into nucleotide sequence types (ntSTs) by using DnaSP version 6.12.03 (Table 2). The validated *Anaplasma* sequences were grouped into four ntSTs and representative sequence from each ntST was submitted to the GenBank database, including ntST1 (37 sequences; Acc. No. OQ692407), ntST2 (three sequences; Acc. No. OQ692408), ntST3 (one sequence; Acc. No. OQ692409), and ntST4 (one sequence; Acc. No. OQ692410) (Table 2). Among four ntSTs of the *Anaplasma* sequences, BLAST results showed ntST1 had 100% identity with various ruminant-related *Anaplasma*, including *A. capra* (ON872236) from horse in Iraq, *A. marginale* (OP851751) from cattle in India, *A. ovis* (OM282854) from sheep in Russia, and *Anaplasma* spp. (KY766240) from *Rhipicephalus microplus* tick in Thailand. The ntST2 shared 100% identity with *Anaplasma* spp. (AF497579) from the *Haemaphysalis lagrangei* tick in Thailand and *A. bovis* (OQ132528) from *H. hystricis* tick in China. The ntST3 and 4 shared 100% identity with the *Anaplasma* spp. (MH589424) from a mountain bongo in Kenya and *A. bovis* (KP062954) from a goat in China, respectively (Table 2).

**Table 2:** Nucleotide sequence types (ntSTs), NCBI BLAST results, and accession number of the representative nucleotide sequences obtained in this study.

ntSTs	Number of sequences (*N* = 67)	Highest BLAST result		Submitted sequences (Acc. No.)
		Closely related species	% Identity	
***Anaplasma* sequences**				
1	37	*A. capra* (ON872236)*, A. marginale* (OP851751)*, A. ovis* (OM282854), and *Anaplasma* spp. (KY766240)	100	OQ692407
2	3	*Anaplasma* spp. (AF497579) and *A. bovis* (OQ132528)	100	OQ692408
3	1	*Anaplasma* spp. (MH589424)	100	OQ692409
4	1	*A. bovis* (KP062954)	100	OQ692410
***Bartonella* sequences**				
5	10	*Bartonella* spp. (LC485116)	99.11	OQ716819
6	1	*Bartonella* spp. (CP019781)	97.65	OQ716820
7	3	*Bartonella* spp. (LC485115)	98.22	OQ716821
8	6	*Bartonella* spp. (LC485116)	98.52	OQ716822
9	1	Uncultured bacterium (JX416234)	97.35	OQ716823
10	2	*Bartonella* spp. (CP019781)	97.65	OQ716824
11	1	*Bartonella* spp. (LC485115)	98.52	OQ716825
12	1	*Bartonella* spp. (LC485115)	97.63	OQ716826

Twenty-five sequences obtained from *Bartonella* PCR-positive samples were grouped into eight ntSTs and representative sequence from each ntST was submitted to the GenBank database, including ntST5 (10 sequences; Acc. No. OQ716819), ntST6 (one sequence; Acc. No. OQ716820), ntST7 (three sequences; Acc. No. OQ716821), ntST8 (six sequences; Acc. No. OQ716822), ntST9 (one sequence; Acc. No. OQ716823), ntST10 (two sequences; Acc. No. OQ716824), ntST11 (one sequence; Acc. No. OQ716825), and ntST12 (one sequence; Acc. No. OQ716826) (Table 2). The BLAST results showed ntST5 and 8 shared the highest similarity to *Bartonella* spp. (LP485116) from deer ked in Japan with 99.11 and 98.52%, respectively. The ntST6 and 10 had the highest similarity to *Bartonella* spp. (CP019781) from sika deer in Japan with 97.65%, while ntST7, 11, and 12 showed the highest similarity to *Bartonella* spp. (LC485115) from deer ked in Japan, with 98.22, 98.52, and 97.63%, respectively. Lastly, ntST9 showed the highest similarity to the uncultured bacterium (JX416234) with 97.35% from a bat fly in the USA (Table 2).

### 3.3. Phylogenetic analysis of *Anaplasma* and *Bartonella* detected in *Lipoptena* specimens

**Figure 1:**
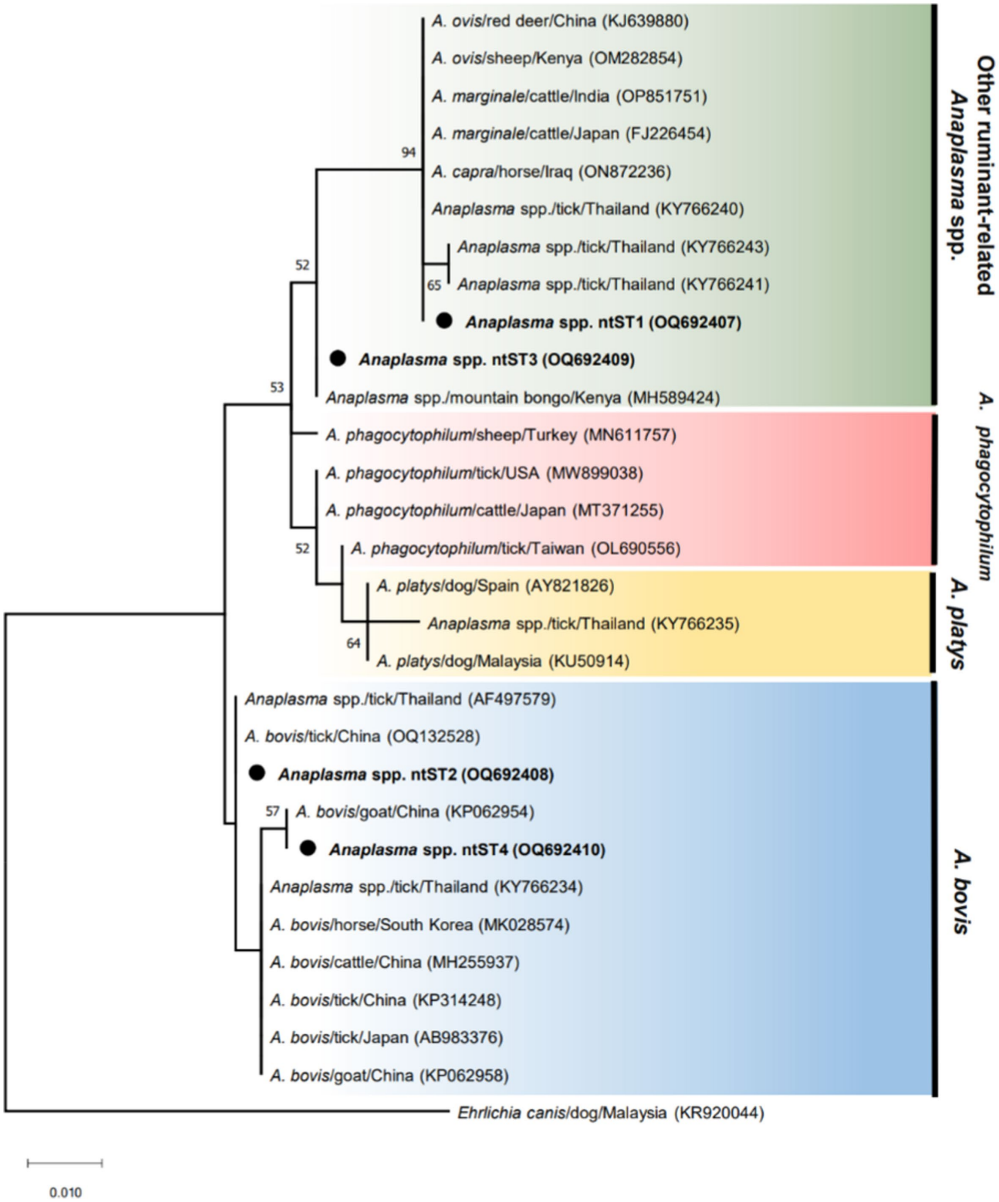
ML tree of 16 s rRNA gene of *Anaplasma* sequences (305 bp) computed with the K2 + G model. The phylogenetic relationships among sequences obtained in this study (black dot) and reference sequences from the GenBank database. *Ehrlichia* sequence isolated from dog (KR920044) represents as an out group.

**Figure 2:**
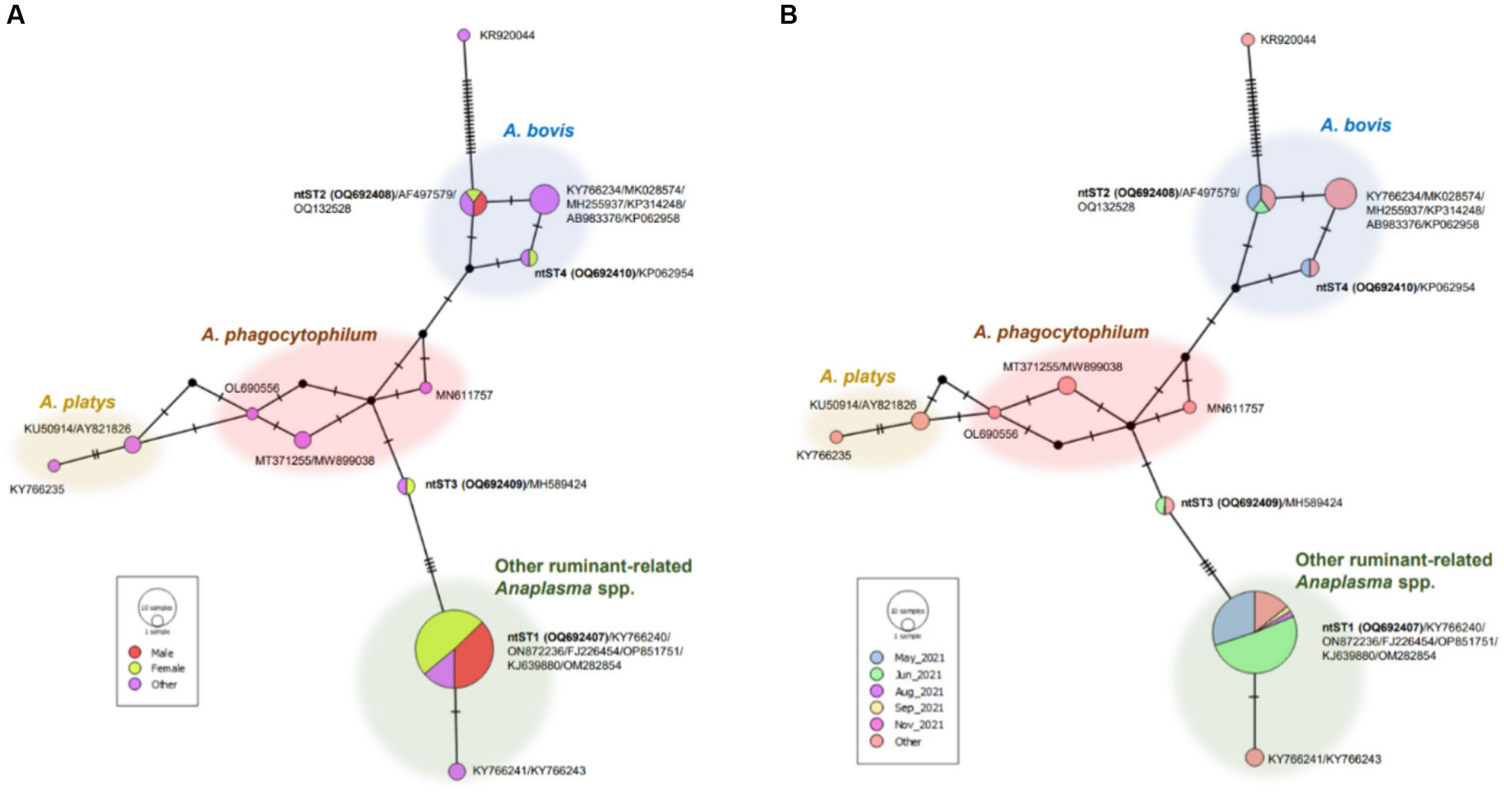
ntST network of 16S rRNA gene of *Anaplasma* spp. ntST1 to ntST4 represented sequences obtained in this study, while other sequences obtained from the GenBank database relating to the reference sequences shown in Figure 1. The size of circle represents the frequency of each ntST, whereas the color represents the gender of specimens **(A)** and collecting date **(B)**. “Other” refers to the reference sequences.

The phylogenetic tree of *Anaplasma* sequences showed samples in ntST2 and 4 clustered in the same clade as *A. bovis*, while samples in ntST1 and 3 grouped with clade of other ruminant-related *Anaplasma* spp. (Figure 1). Figure 2 shows the ntST network of 16S rRNA gene of *Anaplasma* spp. from a total of 12 ntSTs (68 sequences). ntST1 to ntST4 represented *Anaplasma* sequences obtained in this study. From the ntST network, ntST2 and 4 were classified into *A. bovis* group, which differed by one mutation step from ntST of the *A. bovis* clade (KY766234, MK028574, MH255937, KP314248, AB983376, and KP062958) found in ruminants and ticks in several countries (Figure 2). Furthermore, two mutation steps separated ntST2 from ntST4 (Figure 2). The samples in ntST1 were grouped with clade of other ruminant-related *Anaplasma* from GenBank; *Anaplasma* spp. from tick (KY766240), *A. capra* (ON872236), *A. marginale* (FJ226454, OP851751), *A. ovis* (KJ639880, OM282854; Figure 2). The samples in ntST3 were grouped with a sequence from mountain bongo in Kenya (MH589424), which differed by two (MN611757, MT371255, and MW899038) and three (OL690556) mutation steps from the clade of *A. phagocytophilum* (Figure 2).

**Figure 3:**
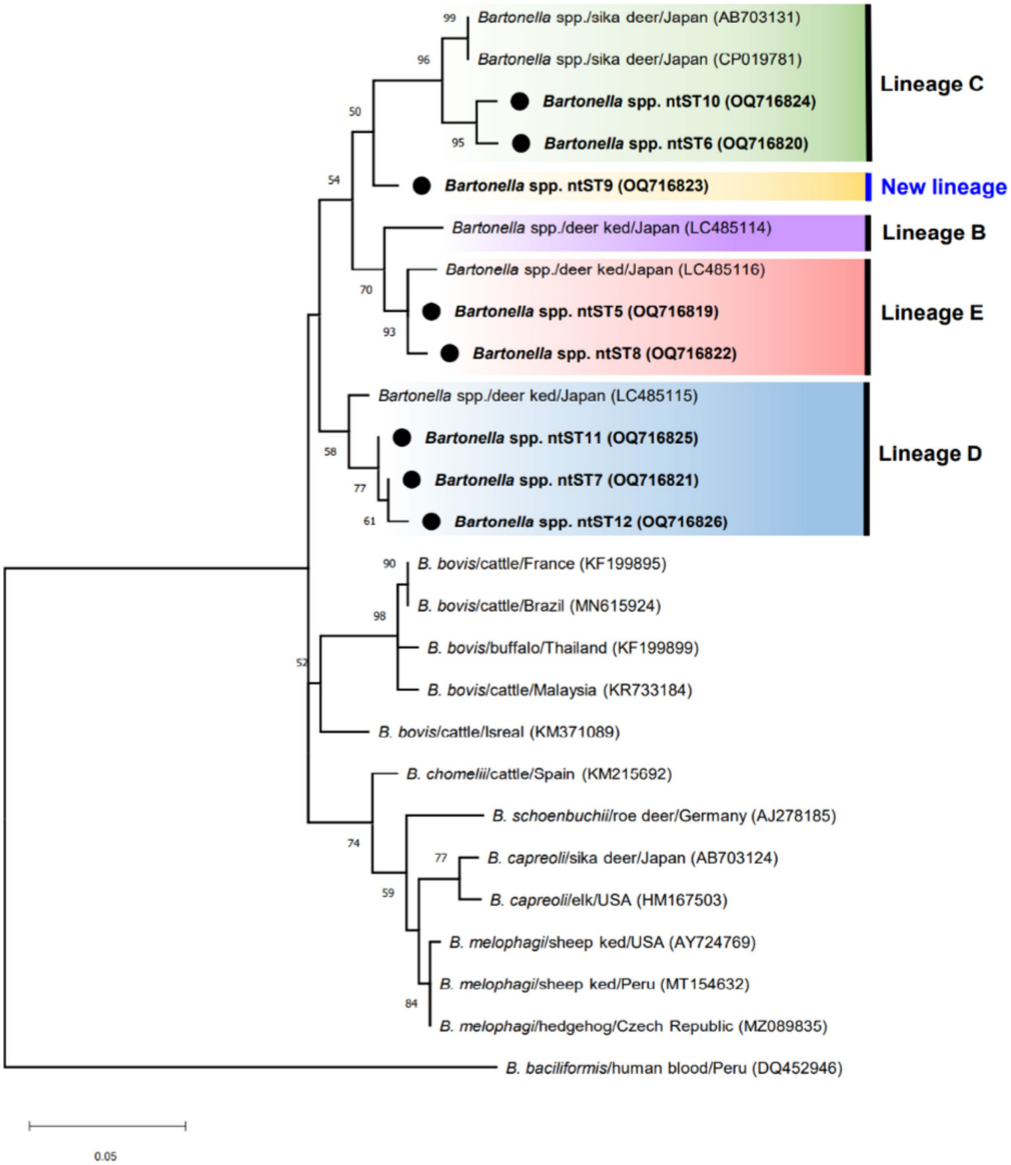
ML tree of *gltA* gene of *Bartonella* sequences (337 bp) computed with the TN93 + G model. The phylogenetic relationships among sequences obtained in this study (black dot) and ruminant-related *Bartonella* sequences from the GenBank database. Lineages B, C, D, and E were determined by Sato et al. (15, 39). *Bartonella bacilliformis* sequence isolated from human (KR920044) represents as an out group.

**Figure 4:**
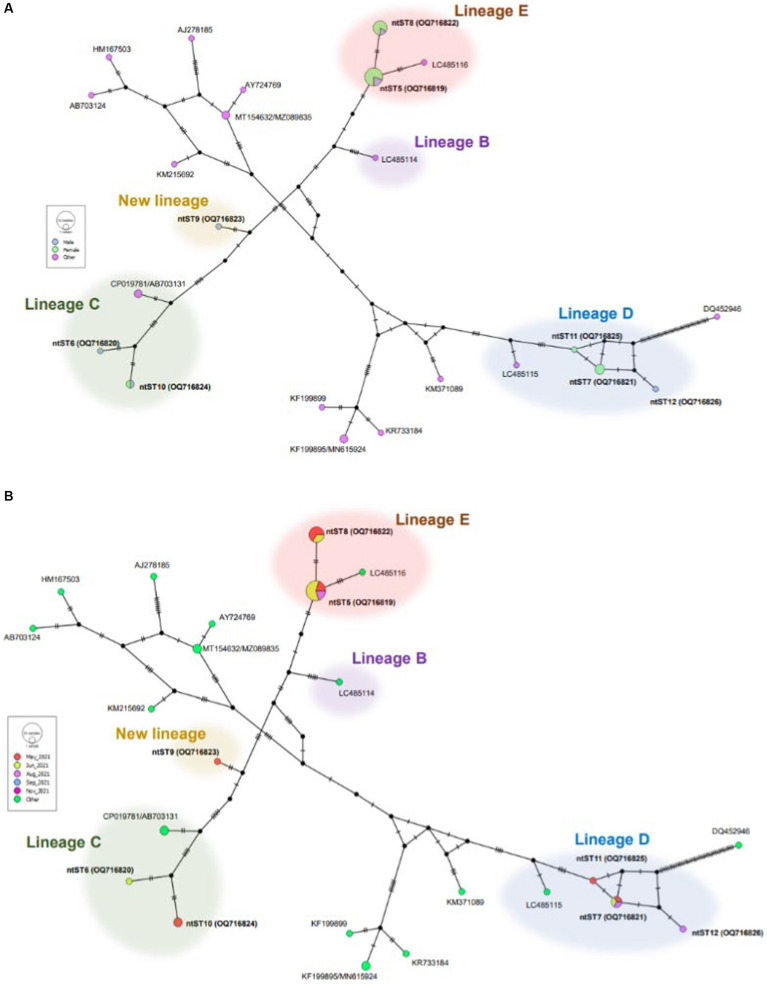
ntST network of *gltA* gene of *Bartonella* spp. ntST5 to ntST12 represented sequences obtained in this study, while other sequences obtained from the GenBank database relating to the reference sequences shown in Figure 3. The size of circle represents the frequency of each ntST, whereas the color represents the gender of specimens **(A)** and collecting date **(B)**. “Other” refers to the reference sequences. Lineages B, C, D, and E were determined by Sato et al. (15, 39).

Figure 3 shows phylogenetic tree of *Bartonella* sequences and ntST5 to 12 referred sequences obtained in this study. Except for the ntST9 that represents a novel *Bartonella* species, all obtained sequences were grouped into a distinct *Bartonella* phylogenic lineages C, D, and E, representing a novel *Bartonella* species (15, 39) (Figure 3). Samples in ntST6 and 10 belong to a distinct phylogenetic branch within lineage C, while lineage D is represented in the current study by samples in ntST7, 11, and 12 (Figure 3). Samples in ntST5 and 8 belong to the distinct phylogenetic lineage E (Figure 3). Notably, the phylogenic branch of ntST9 was separated from the clade of lineage C, representing a new independent lineage of a novel *Bartonella* species (Figure 3). The ntST network of *gltA* gene of *Bartonella* spp. from a total of 23 ntSTs (32 sequences) was showed in Figure 4. Based on the distinct phylogenic lineages of a novel *Bartonella* spp., the ntSTs of the *Bartonella* sequences obtained in this study could divided into three lineages: C (ntST6 and 10), D (ntST7, 11, and 12), and E (ntST5 and 8; Figure 4). Both ntST6 and 10 differed by eight mutation steps from clade of lineage C found from sika deer in Japan (CP019781 and AB703131; Figure 4). The ntST7, 11, and 12 were separated from the clade of lineage D found in *L. fortisetosa* collected in Japan (LC485115) by six, five, and eight mutation steps, respectively (Figure 4). The samples in ntST5 differed by three mutation steps from clade of lineage E found in *L. fortisetosa* collected in Japan (LC485116), while samples in ntST8 differed by five mutation steps (Figure 4). Furthermore, ntST9, respectively, differed from the designated novel *Bartonella* sequence lineages B, C, D, and E by 12, 10, 18, and 13 mutation steps, which suggests a new independent lineage of a novel *Bartonella* species (Figure 4).

## 4. Discussion

*Lipoptena fortisetosa* is a crucial ectoparasite infesting cervids worldwide. This insect is primarily found in sika deer (*Cervus nippon*) in Japan and has also been reported in Siberian roe deer (*Capreolus pygargus*) in Korea, Kazakhstan, and Russia (1, 9, 52). The distribution of *L. fortisetosa* in European countries has been hypothesized by climate change, introduction of alien cervid species into new areas, and adaptation of the insect to different hosts (11, 12). In Thailand, *L. fortisetosa* was first found on captive Eld’s deer in Chon Buri, as previously reported (44). *Lipoptena* insects may cause anemia, skin irritation, itching, restlessness, and hair loss in animal hosts (53). However, there were no skin or other symptoms on infested Eld’s deer in Thailand.

To our knowledge, this is the first report on *Anaplasma* and *Bartonella* detection in *L. fortisetosa* in Thailand. The prevalence of *Anaplasma* spp. detection in *L. fortisetosa* (46.15%) in the present study was higher than that reported in Poland (8.00%) (54). Although no previous evidence exists of *Anaplasma* harbored by this insect in the country, the presence of ticks, the primary *Anaplasma* vector, in the same area of wildlife habitat may enhance the possibility of bacterial infection in other blood-feeding ectoparasites, including *Lipoptena* insects. This possibility is supported by the results where ntST2 was identical to *Anaplasma* detected in *H. lagrangei* ticks in a previous study (43). In addition to *Anaplasma*, various pathogens, such as *Babesia*, *Ehrlichia*, *Theileria*, and *Wolbachia*, have also been detected in questing ticks in wildlife habitat in Chon Buri, Thailand (43). This finding highlights that, in the same surrounding area, various ectoparasites may harbor, or transmit the pathogen. Moreover, *Anaplasma* DNA is found in other species of *Lipoptena* insects, such as *L. cervi* and *L. depressa* (19, 23, 55).

*Anaplasma* DNA fragments from this study can be clustered with the clades of *A. bovis* and other ruminant-related *Anaplasma*. *A. bovis* has previously been detected in domestic goats (56), but no report on wildlife in Thailand. Interestingly, *A. bovis* DNA has been found in ticks collected from the Malayan sun bear (*Helarctos malayanus*), sambar deer (*Cervus unicolor*), and questing ticks dragged in wildlife habitat in Thailand (25, 43, 47). These findings suggest wild animals may act as a natural reservoir and their ectoparasites may be associated with *Anaplasma* infection of domestic ruminants. This possibility is supported by the present study showing ntST2 and 4 showed similar genetic material with *A. bovis* detected in goats in China (OQ132528 and KP062954, respectively). Other ruminant-related *Anaplasma*, including *A. marginale*, *A. ovis*, and *A. capra*, have been detected in various wild animals, suggesting a broad host range and genetic diversity (57–59). In the present study, using primers for the partial 16S rRNA gene of *Anaplasma* did not distinguish obtained sequence data from other ruminant-related *Anaplasma*. Amplification and sequencing of full-length 16S rRNA gene or other specific genes of *Anaplasma* would be necessary to clarify genetic characterization.

Besides biological transmission of *Anaplasma* by ticks, other potential vectors have been reported to mechanically transfer bacteria to animal hosts, including biting flies (60) and syringophilid mites (61). For *Lipoptena*, the insects detach their wings after finding suitable hosts and can only be transferred among hosts via direct contact. Since the insects acquire *Anaplasma*-infected blood meal on bacteremic hosts, it is possible to transmit bacteria horizontally to noninfected animals via direct contact (55). However, further experimental studies are needed to confirm direct evidence of the vector ability for *Anaplasma* bacteria transmission.

The prevalence of *Bartonella* detection in *L. fortisetosa* (29.67%) in this study was lower than those collected from free-living cervids in Japan (87.87%) and Poland (75.67%) (15, 62). Possible reasons for low prevalence of *Bartonella* detection are that Eld’s deer are raised in captive areas in Thailand. The semi-wild conditions of deer may confer a lower infestation probability by pathogen-infected ectoparasites compared with free-living wild cervids in Japan and Poland. This possibility is supported by a previous study in Poland showing farm cervids had a lower prevalence of *A. phagocytophilum* infection than wild individuals (21). Myczka et al. (21) also mentioned the lack of *Anaplasma* detection in farmed cervids may be due to regular deworming, which strengthens their condition and makes them less susceptible to infection by the bacteria. Notably, novel *Bartonella* has been detected and isolated with low prevalence (3.60%) from captive Rusa deer in Thailand, including those being regularly dewormed (40). Secondly, despite prior tick presence in the same surrounding area (43), ticks may not be the essential vector for *Bartonella* transmission among cervids (15, 63). For this reason, we implied that since Eld’s deer are infested by ticks and *Lipoptena* insects, *Bartonella* can still be detected, merely not in high prevalence. However, *Bartonella* detected in captive Eld’s deer and ticks collected from surrounding areas should be analyzed to clarify these reasons.

*Bartonella* sequences obtained from this study can be grouped with novel *Bartonella* lineages C, D, and E, which originated from *L. fortisetosa* collected from deer in Japan (15, 39). In addition, the novel *Bartonella* lineage B, primarily derived from Japanese sika deer, has previously been found in *L. cervi* and *L. fortisetosa* collected from red deer in Poland (62, 64). These findings suggest novel *Bartonella* stains from wild ruminants in Japan may spread to other countries following *Lipoptena* vector introduction into these new areas. We also found a new independent lineage of novel *Bartonella* from collected *Lipoptena* insects. However, genetic characterization analysis is needed to determine whether these new lineages are *Lipoptena* insects-specific *Bartonella*. Obtained *Bartonella* sequences were clustered with ruminant-related *Bartonella*. In addition to the report of novel *Bartonella* isolated from captive Rusa deer in Thailand (40), further reports exist of *Bartonella* detected or isolated from domestic ruminants in the country. Bai et al. (65) revealed that *B. bovis* was isolated from water buffalo blood. In 2021, seroprevalence of antibodies against *B. henselae*, *B. vinsonii* subspp. *Berkhoffii*, and *B. tamiae* in water buffaloes has also been reported (66). These findings support the genetic diversity of *Bartonella* among ruminants in Thailand. Further molecular surveys of *Bartonella* in both wild and domestic ruminants and their ectoparasites are needed to clarify the role of bacterial infection among ruminants in the country.

The high prevalence of *Bartonella* DNA presence in *Lipoptena* insects raises the question that insects may play an essential role in *Bartonella* transmission among hosts (5, 15, 34, 37, 38). The evidence of *Bartonella* survival and propagation in *Lipoptena* specimens has been reported from previous studies using bacterial isolation from the insects and immunohistochemical analysis (15, 35). In addition, the bacterial DNA detected in both wingless *L. cervi* females and their offspring suggests the potential for vertical *Bartonella* transmission (34). However, both *in vitro* and *in vivo* studies are required to verify how vector competence of *Lipoptena* insects facilitates *Bartonella* transmission.

Co-infections occur in *Lipoptena* insects but pathogen diversity may vary by species, hosts, and geographic distribution. In the USA, 6.25% of *L. cervi* removed from white-tailed deer carried both *B. burgdorferi* s.l. and *A. phagocytophilum* DNA (19). Further, 50% of *L. fortisetosa* collected from Korean water deer harbored *Coxiella*, *T. ovis*, and *T. luwenshuni* DNA, but no *Rickettsia*, *Babesia*, *Bartonella*, *Borrelia*, or *Hepatozoon* were detected (67). In this study, 11 *Lipoptena* specimens (12.08%) harbored both *Anaplasma* and *Bartonella* DNA. We also found four specimens were additionally infected with *Theileria* spp. as previously reported by Tiawsirisup et al. (44). The prevalence of more than one pathogen in *Lipoptena* insects emphasizes their medical and veterinary importance.

In the present study, our findings provide the first molecular detection of *Anaplasma* and *Bartonella* on *L. fortisetosa* in Eld’s deer in the country. Despite no blood samples from Eld’s deer, pathogen DNA detected in insects could represent the health status of animal hosts. Further studies on molecular genetic characterization of related pathogens are needed to investigate correlations of vectors, hosts, and pathogens. In addition, visitors are allowed to have direct contact with animals through petting, feeding, or taking close photos with animals in the zoo. These activities can promote the risks of potential zoonotic infection. Preventive procedures, such as health monitoring, anti-parasite medication, and proper treatments for animals should be regularly conducted. Zoo staff should pay attention to regular hygiene measures before and after working with animals and surrounding areas, such as hand washing, wearing PPE, and foot bathing with an antiseptic solution. Finally, visitors should avoid direct contact with animals and be wary of insects or ectoparasites while visiting the zoo.

## Data availability statement

The datasets presented in this study can be found in online repositories. The names of the repository/repositories and accession number(s) can be found at: https://www.ncbi.nlm.nih.gov/, OQ692407 https://www.ncbi.nlm.nih.gov/, OQ692408 https://www.ncbi.nlm.nih.gov/, OQ692409 https://www.ncbi.nlm.nih.gov/, OQ692410 https://www.ncbi.nlm.nih.gov/, OQ716819 https://www.ncbi.nlm.nih.gov/, OQ716820 https://www.ncbi.nlm.nih.gov/, OQ716821 https://www.ncbi.nlm.nih.gov/, OQ716822 https://www.ncbi.nlm.nih.gov/, OQ716823 https://www.ncbi.nlm.nih.gov/, OQ716824 https://www.ncbi.nlm.nih.gov/, OQ716825 https://www.ncbi.nlm.nih.gov/, OQ716826.

## Ethics statement

The animal study was approved by the Chulalongkorn University Animal Care and Use Committee (Animal use protocol number: 2231011). The study was conducted in accordance with the local legislation and institutional requirements.

## Author contributions

WW contributed data, performed the analysis, and wrote the manuscript. CS-i and KT collected the data and provided technical support. NY, CA, GR, NB, and NS collected samples and data. LB edited the manuscript. UM and PK provided professional support at the sample collecting sites. AS provided technical support. ST supervised the study, edited the manuscript, and corresponded. All authors contributed to the article and approved the submitted version.

## Funding

This research was funded by the Thailand Science Research and Innovation (TSRI) Fund (CU_FRB640001_01_31_3), the Chulalongkorn University Research Unit (GRU 3310160009), and the Second Century Fund (C2F), Chulalongkorn University.

## Conflict of interest

The authors declare that the research was conducted in the absence of any commercial or financial relationships that could be construed as a potential conflict of interest.

## Publisher’s note

All claims expressed in this article are solely those of the authors and do not necessarily represent those of their affiliated organizations, or those of the publisher, the editors and the reviewers. Any product that may be evaluated in this article, or claim that may be made by its manufacturer, is not guaranteed or endorsed by the publisher.
